# Prospective Associations of Erythrocyte Composition and Dietary Intake of *n*-3 and *n*-6 PUFA with Measures of Cognitive Function

**DOI:** 10.3390/nu10091253

**Published:** 2018-09-06

**Authors:** Sherman J. Bigornia, Tammy M. Scott, William S. Harris, Katherine L. Tucker

**Affiliations:** 1 Department of Agriculture, Nutrition, and Food Systems, University of New Hampshire, Durham, NH 03824, USA; 2 USDA Jean Mayer Human Nutrition Research Center on Aging, Tufts University, Boston, MA 02111, USA; Tammy.Scott@tufts.edu; 3 Medicine, Sanford School of Medicine, University of South Dakota, Sioux Falls, SD 57105, USA; Bill@omegaquant.com; 4 Omegaquant, LLC, Sioux Falls, SD 57105, USA; 5 Department of Biomedical and Nutritional Sciences, University of Massachusetts Lowell, Lowell, MA 01854, USA; Katherine_Tucker@uml.edu

**Keywords:** omega-3 fatty acids, omega-6 fatty acids, cognitive function, Boston Puerto Rican Health Study, erythrocyte fatty acids

## Abstract

Polyunsaturated fatty acid (PUFA) consumption is recommended as part of a healthy diet, but evidence of the impact of individual species and biological concentrations on cognitive function is limited. We examined prospective associations of PUFA erythrocyte composition and dietary intake with measures of cognitive function among participants of the Boston Puerto Rican Health Study (aged 57 years). Erythrocyte and dietary PUFA composition were ascertained at baseline and associated with 2-year scores on the Mini-Mental State Exam (MMSE) (*n* = 1032) and cognitive domain patterns derived from a battery of tests (*n* = 865), as well as with incidence of cognitive impairment. Erythrocyte and dietary *n*-3 PUFA were not significantly associated with MMSE score. However, total erythrocyte and dietary *n*-3 very-long-chain fatty acids (VLCFA), and intake of individual species, were associated with better executive function (*P*-trend < 0.05, for all). There was evidence that greater erythrocyte *n*-6 eicosadienoic acid concentration was associated with lower MMSE and executive function scores (*P*-trend = 0.02). Only erythrocyte arachidonic acid (ARA) concentration predicted cognitive impairment (Odds Ratio = 1.26; *P* = 0.01). Among Puerto Rican adults, we found that *n*-3 VLCFA consumption may beneficially impact executive function. Further, these findings provide some evidence that *n*-6 metabolism favoring greater ARA tissue incorporation, but not necessarily dietary intake, could increase the risk of cognitive impairment.

## 1. Introduction

With the continued growth of the elderly population, estimated to reach 17% globally by 2050 [1], the number of individuals living with cognitive decline and dementia is expected to increase. Cognitive decline can impair one’s ability to make decisions [2], and reduce quality of life and independence [3,4]. Neurological conditions are the third leading cause of years lived with disability [5].

Limited evidence suggests that Latinos may experience disproportionate cognitive impairment [6,7,8,9,10,11]. Cross-sectional data from the Hispanic Community Health Study/Study of Latinos suggest that Caribbean Latinos, specifically Puerto Ricans and Dominicans, may have twice the odds of low cognitive status compared to Mexican Americans [12]. Caribbean Latinos, compared to those of other Latino descent, appear to have greater presence of risk factors for dementia (e.g., hypertension, insulin resistance, inflammation, obesity, dyslipidemia) [13,14,15,16,17]. The apparent greater risk of cognitive impairment among Latinos, particularly Caribbean Latinos, calls for a better understanding of modifiable risk factors relevant to the Latino population.

The human brain is predominantly composed of lipids, and a large proportion of the polyunsaturated fatty acids (PUFA) are *n*-3 PUFA, specifically docosahexaenoic acid (DHA) [18]. A meta-analysis of prospective cohort studies found that *n*-3 PUFA consumption was inversely related to risk of dementia and Alzheimer’s disease [19]. However, these results were not supported by another meta-analysis published around the same time [20]. Erythrocyte fatty acid (FA) concentrations offer a long-term measure of *n*-3 exposure (past 3 months) and status. However, only a handful of prospective studies have examined erythrocyte FA composition [21], with some, but not all [22,23], suggesting a protective association against decline in cognitive function or development of dementia [24,25].

PUFA consumption, including *n*-6 PUFA, are recommended as part of a healthy diet for cardiovascular health [26]. Compared to *n*-3 PUFA, there is less evidence available regarding the potential influence of n-6 PUFA consumption and metabolism on cognitive function. One study reported that higher erythrocyte total *n*-6 PUFA concentration was adversely associated with cognitive function [24]. However, *n*-6 linoleic acid (LA) status has been related to lower risk of type 2 diabetes [27] and coronary heart disease [28,29], chronic conditions thought to increase the risk of cognitive impairment. Thus, it is necessary to conduct prospective studies of the associations of biological PUFA concentrations and dietary intake with longitudinal measures of cognitive function, to clarify the impact of *n*-3 and *n*-6 PUFA consumption and metabolism on these health outcomes.

We sought to quantify the associations of individual erythrocyte and dietary *n*-3 and *n*-6 PUFA with 2-year general cognitive function (mini-mental state exam score, MMSE) and specific cognitive function domains derived from a battery of cognitive tests in a prospective cohort of older Puerto Rican Adults living on the US mainland. In addition, the ability of individual PUFA species to predict 2-year incidence of cognitive impairment was investigated.

## 2. Materials and Methods

### 2.1. Participants

We used data from the Boston Puerto Rican Health Study (BPRHS), a longitudinal cohort designed to examine sociological, environmental, and genetic factors that influence quality of life and risk for chronic diseases in Puerto Rican adults living in the greater Boston, MA area [30]. Recruitment procedures utilized information from the 2000 Census by identifying tracks with at least 25 Puerto Rican adults and, within these, randomly selecting census blocks of ≥10 Hispanic adults, which were enumerated by home visits to identify eligible participants. While most were recruited in this manner [30], some participants were recruited by alternative means, such as random approach at cultural events or personal referral. Recruitment occurred from 2004 to 2009. Participants included those who self-identified as Puerto Rican, and who were English or Spanish speaking. Individuals were excluded if considered to have severe cognitive impairment at baseline, as determined by a MMSE score of <10. The baseline cohort included 1500 individuals (45–75 years) and cited reasons for non-participation were as previously described [30]. Information on potential confounders was available for 1404 participants, of which 1197 returned for a 2-year follow-up visit. We excluded those missing baseline erythrocyte FA composition (*n* = 127). In the examination of general cognitive function, we excluded those individuals missing 2-year MMSE values (*n* = 38), for an analytical sample size of 1032. For sets of analyses examining cognitive function patterns, models excluded those that did not have both baseline (*n* = 113) and 2-year (*n* = 114) cognitive battery tests, resulting in a sample size of 865.

The protocol for this study was approved by the Institutional Review Boards at Tufts Medical Center, Northeastern University, and the University of Massachusetts Lowell. Written informed consent was obtained from all participants and all study-related activities were conducted according the guidelines laid down in the Declaration of Helsinki.

### 2.2. Erythrocyte Membrane PUFA Composition

Participants were asked to fast for 12 h preceding the blood draw, which was obtained in-home by a certified phlebotomist. Blood samples were centrifuged to obtain plasma and the erythrocyte pellet. Aliquots were stored at −70 °C for later use [30]. As previously described [31], erythrocyte FA composition was ascertained by gas chromatography with flame ionization detection (GC2010, Shimadzu Corp., Columbia, MD, USA). Individual FA were expressed as a percentage of total identified FA. Available *n*-3 and *n*-6 PUFA species were examined (Supplemental Table S1). The coefficients of variation ranged from 0.13% to 16.6% (Supplemental Table S1). Study participants were categorized using quartiles according to individual erythrocyte PUFA concentration (Supplemental Table S2).

### 2.3. Diet and Dietary PUFA Assessment

Self-reported dietary intake from the previous 12 months was obtained by trained interviewers using a semi-quantitative food frequency questionnaire (FFQ) that had been modified and validated for use in the Puerto Rican population [32]. Nutrient intakes were determined using the Nutrient Data System for Research software, version 11 (NDS-R, Nutrition Coordinating Center, Minneapolis, MN, USA). A more recent iteration of NDS-R (version 16, Minneapolis, MN, USA) was used to estimate α-linolenic acid (ALA) consumption because it was not available in the 2011 version. Individual available PUFAs were expressed as a percentage of total energy and categories of intake were defined using quartiles (Supplemental Table S2).

### 2.4. Assessment of Cognitive Function

Trained bilingual interviewers administered a full battery of seven cognitive tests to BPRHS participants during in-home visits in either Spanish (98%) or English, as preferred by the participant [33,34]. General cognitive function was quantified using the MMSE [35]; scores in this sample ranged from 12 to 30. Participants were characterized as having mild or greater cognitive impairment using educational level-specific MMSE score cut-offs [30,33] defined as MMSE score <21 for those with less than a high school education or General Education Development certificate (GED), <23 for those that completed high school or GED certification, and <24 for those with some college education.

A battery of cognitive tests was administered, and included Word List Learning [36], Digit Span Forward and Backward [36], the Stroop Test [36], Verbal Fluency [36], Clock Drawing [37], and Figure Copying [38] (Supplemental Table S3). Higher scores on each test signify better cognitive functioning. Since there is some redundancy in the cognitive domains assessed by each of these tests, we performed principal components analysis (PCA) using the scores from the seven cognitive tests (not including MMSE) [33,34]. Factors were orthogonally rotated using the varimax option to maximize explanatory power and to produce uncorrelated factors. We started with 2 to 7 factor solutions, and inspected scree plots, eigenvalues, and individual factors for interpretability [39]. Two factors were retained and were interpreted as representing executive function (factor 1) and memory (factor 2) domains (Supplemental Table S3).

### 2.5. Covariates

Sociodemographic and medical information was collected by self-reported questionnaire, including age, sex, smoking status, educational attainment, medical history, and medication use. Smoking was categorized as current or not currently smoking at baseline. Education was categorized as high school educated (yes or no) if they had received either a high school diploma or GED certification. Those presenting with a baseline fasting blood sugar ≥126 mg/dL or taking an antihyperglycemic medication were classified as having diabetes. Participants reporting whether a doctor had ever told them that they have had a heart attack, heart disease (other than a heart attack), or stroke were considered to have heart disease. 

Physical activity level was assessed by a modified Paffenbarger questionnaire from the Harvard Alumni Activity Survey [40,41], which has been used previously in an elderly Puerto Rican population [42]. A physical activity score was calculated as the sum of hours spent on typical 24-h activities (heavy, moderate, light, or sedentary activity, and sleeping) multiplied by weighting factors that parallel the rate of oxygen consumption associated with each activity.

Depressive symptomatology was assessed using the Center for Epidemiologic Studies Depression scale (CES-D). The CES-D includes 20-items with questions referring to symptoms during the week prior to the interview and scores range from 0 to 60 [43,44,45]. Clinically elevated depressive symptomatology was defined as a CES-D score ≥16 [46].

As described elsewhere [33], the Healthy Eating Index (HEI) 2005 score was calculated using procedures consistent with those from the USDA Center for Nutrition Policy and Promotion [47]. The HEI 2005 score was chosen because it is consistent with US dietary recommendations at the time of dietary assessment [48]. The HEI 2005 score includes 12 components (e.g., oils, total fruit, total vegetables, whole grains, and energy from solid fats, alcoholic beverages, and added sugars). The oils component includes oils from non-hydrogenated vegetable oils, and those found in fish, nuts, and seeds [47]. To capture variation in dietary quality not due to these oils, we adjusted models using a modified HEI 2005 score that excluded the oil component.

Applied Biosystems’ TaqMan single nucleotide polymorphism genotyping procedures [49,50] were used to ascertain ApoE genotype. This information was available for most participants (*n* = 756). ApoE ε4 genotype was defined dichotomously as the presence or absence of the ε4 allele.

### 2.6. Statistical Analyses

We considered our analyses in several stages. First, differences in sample characteristics across quartiles of *n*-3 and *n*-6 PUFA erythrocyte composition and dietary intake were examined by ANCOVA, and adjusted for age and sex, as appropriate. Associations between erythrocyte and dietary PUFA species were examined using Spearman’s correlation coefficients adjusted for age, sex, and energy intake. Second, we investigated whether erythrocyte composition and dietary intake of individual *n*-3 and *n*-6 PUFA species were associated with changes in global cognitive function (MMSE score) over the 2-year follow-up. We also examined the impact of *n*-3 very-long-chain FA (VLCFA) status and dietary intake, which was calculated as the sum of eicosapentaenoic (EPA), docosapentaenoic (DPA), and DHA. N-3 VLCFAs are products of ALA metabolism, and whereas ALA is primarily found in plants, *n*-3 VLFCAs are concentrated in animal food sources (e.g., fatty cold-water fish). Erythrocyte and dietary PUFA categories were defined using quartiles, and 2-year MMSE score was expressed as a continuous variable. Differences in MMSE score across PUFA categories were examined using ANCOVA with post hoc comparisons conducted by Tukey tests, with the reference category set as the lowest quartile. Models were adjusted for baseline MMSE score and time between visits, as well as for other covariates, described previously. For models examining *n*-3 PUFA as the exposure, *n*-6 linoleic acid (LA) and *n*-6 arachidonic acid (ARA) were also included as covariates, whereas *n*-6 PUFA models were adjusted for *n*-3 ALA and *n*-3 VLCFA. This was done to account for the dependency of essential FA *n*-3 ALA and *n*-6 LA metabolism upon one another, due to shared enzymes that perform the elongation and desaturation steps necessary to generate longer-chain FA in both pathways [51,52]. Adjusted mean MMSE scores were estimated using ANCOVA, and tests for linear trend were conducted by treating PUFA categories (quartile) as a continuous variable. A similar set of analyses were conducted with 2-year executive function domain or memory domain pattern score as the outcome variables. All associations with the memory scores did not attain statistical significance. Thus, only results with the executive function domain are presented. Third, the ability of individual *n*-3 and *n*-6 PUFA to predict 2-year incidence of cognitive impairment was measured using logistic regression. These analyses were restricted to those participants without baseline cognitive impairment (*n* = 688). Models were constructed in a similar fashion as described for those examining 2-year MMSE score and PCA-derived cognitive patterns, but models were not adjusted for baseline cognitive testing score. Lastly, we also considered several additional analyses. Most *n*-3 supplement users were in the highest and lowest quartiles of erythrocyte composition and dietary intake of total *n*-3 and *n*-6 PUFA, respectively (Table 1 and Supplemental Table S4). In sensitivity analyses, we repeated all analyses excluding baseline *n*-3 supplement users to determine whether observed associations were driven by these few study participants. In a separate set of analyses, we also adjusted for ApoE ε4 genotype among those 756 participants with available data. To complement analyses with erythrocyte *n*-3 VLCFA as the dependent variable, we also examined the omega-3 index (sum of erythrocyte EPA and DHA). EPA and DHA have been cross-sectionally associated with lower cerebral brain volume and poorer cognitive test scores [53] and lower omega-3 index values appear to be a risk factor for coronary heart disease [54]. Statistical analyses were conducted using SAS version 9.4 (SAS Institute Inc., Cary, NC, USA) with a significance level set at *P* ≤ 0.05.

## 3. Results

### 3.1. Sample Characteristics

A total of 1032 participants had erythrocyte FA composition available at baseline and cognitive function measures at baseline and 2-year follow-up. Sample characteristics varied by baseline *n*-3 and *n*-6 PUFA (Table 1 and Supplemental Table S4).

Values for baseline age, high school educational attainment, HEI score, and *n*-3 supplement use were positively correlated with total erythrocyte *n*-3 composition, but negatively with total *n*-6 composition. Greater *n*-3 composition was associated with lower CES-D score and likelihood of current smoking, but with higher prevalence of cardiovascular disease. The prevalence of diabetes decreased with increasing *n*-6 composition. Dietary *n*-3 PUFA was associated with greater HEI score (with and without oil component), diabetes, and *n*-3 supplement use (Supplemental Table S4). Older age, greater HEI score (with oil component), and lower use of omega-3 supplements were associated with greater dietary *n*-6 PUFA. Correlations between erythrocyte composition and consumption of *n*-3 PUFA, particularly EPA and DHA, were stronger as compared to *n*-6 PUFA (Supplemental Table S5). Correlation coefficients among dietary *n*-3 VLCFA were very strong (rho = 0.84 to 0.93). Further, dietary *n*-3 VLCFA tended to be more strongly correlated with dietary ARA as compared to LA.

### 3.2. MMSE and Executive Function Scores at 2-Year Follow-Up

Erythrocyte and dietary *n*-3 PUFAs were not significantly associated with 2-year MMSE score, when adjusted for baseline MMSE score (Table 2). However, there was a suggestion of positive associations with erythrocyte ALA (*P*-trend = 0.07), dietary EPA (*P*-trend = 0.09), and *n*-3 VLCFA (*P*-trend = 0.08). Higher erythrocyte *n*-3 VLCFA and dietary EPA, DPA, DHA, and dietary *n*-3 VLCFA were positively associated with 2-year executive function score (*P*-trend < 0.05 for all, Table 2). Erythrocyte DHA tended to be associated with higher 2-year executive function score (*P*-trend = 0.06).

Most individual erythrocyte and dietary *n*-6 PUFAs were not significantly associated with either 2-year MMSE or executive function scores, adjusted for baseline scores (Table 3). There was evidence that greater erythrocyte EDA concentration was related to lower MMSE (23.8 Q1 vs. 23.2 Q4, *P*-trend = 0.02) and executive function scores (0.14 Q1 vs. 0.031 Q4, *P*-trend = 0.02). Greater GLA (γ-linolenic acid) concentration tended to be associated with higher MMSE scores (23.4 Q1 vs. 23.8 Q4, *P*-trend = 0.09).

### 3.3. Incidence of Cognitive Impairment

At baseline, 33.3% of participants were considered to have some cognitive impairment according to educational level-specific MMSE cut-offs. Those considered to have cognitive impairment at baseline (vs. not) had higher values for age, CES-D score, and were more likely to be female, and had lower physical activity score, HEI score, and *n*-3 supplement use (Supplemental Table S6). Among those considered to not have cognitive impairment at baseline (*n* = 688), 22% were categorized as having cognitive impairment at the 2-year follow-up visit.

Erythrocyte and dietary *n*-3 PUFA were not significantly associated with 2-year incidence of cognitive impairment (Table 4). The majority of individual *n*-6 PUFA were not significantly related to cognitive impairment (Table 5). However, erythrocyte ARA was associated with a 26% greater risk of cognitive impairment per 1-SD increase [Odds Ratio (OR) = 1.26, *P* = 0.01, Table 5].

### 3.4. Additional Analyses

In general, exclusion of *n*-3 supplement users (2.7%) did not alter our findings, although the association between dietary *n*-3 DPA and 2-year executive function was attenuated (0.04 Q1 vs. 0.18 Q4, *P*-trend = 0.06) (data not shown). Results remained similar after additional adjustment for ApoE ε4 status (data not shown). Compared to models with erythrocyte *n*-3 VLCFA set as the dependent variable, omega-3 index associations with 2-year MMSE (23.2 Q1 vs. 23.4 Q4, *P*-trend = 0.50), executive function score (0.026 Q1 vs. 0.14 Q4, *P*-trend = 0.02), and incident cognitive impairment (OR = 0.90, *P* = 0.26) were similar.

## 4. Discussion

In this prospective cohort study of older Puerto Rican adults, we observed differing associations of erythrocyte and dietary *n*-3 and *n*-6 PUFA species with 2-year global cognitive and executive function, as well as with incident cognitive impairment. In our final models adjusted for baseline cognitive scores, erythrocyte and dietary *n*-3 VLCFA, as well as individual dietary VLCFA species (EPA, DPA, and DHA), were favorably associated with 2-year executive function, but not with 2-year global cognitive function or cognitive impairment. Conversely, *n*-6 EDA was adversely associated with both 2-year global cognitive function and executive function. Further, *n*-6 ARA was associated with a 26% elevated risk of cognitive impairment at the 2-year follow-up visit.

Both circulating concentration and dietary intake of *n*-3 VLCFA, but not ALA, were associated with 2-year executive function scores. This suggests that dietary consumption of *n*-3 VLCFA may have a greater impact on executive function as compared to ALA. Through a series of elongation and desaturation reactions, ALA can be metabolized to EPA and then to DPA, terminating with DHA [52]. EPA and DHA are precursors of anti-inflammatory eicosanoids [55,56], and are associated with lower inflammatory burden [57], blood pressure [58], and improved vascular function [59], all risk factors for cognitive decline. Though DPA has been less studied than EPA and DHA, circulating DPA concentration has been shown to predict lower risk of stroke [60] and type 2 diabetes [61]. Our observed null associations with status and dietary consumption of ALA with executive function, may be partly due to the low rate of conversion of *n*-3 ALA to the more bioactive VLCFA [62]. However, it is worth noting that ALA consumption has been associated with reduction in cardiovascular disease risk [63].

Several randomized controlled trials support the hypothesis that *n*-3 supplementation has beneficial effects against cognitive decline among cognitively normal adults (MMSE > 27) [64,65] or those with mild cognitive impairment or mild Alzheimer’s Disease [66,67]. For example, after 26 weeks of EPA plus DHA supplementation or placebo in older adults (50–75 year), the treatment group improved significantly in executive function, but not in memory, attention, or sensorimotor speed [64]. Further, in a secondary analysis of a 36-week RCT, those participants with baseline omega-3 index values ≤4.8% (lowest quartile) experienced less decline in executive function as measured by the Controlled Oral Word Association Test, but not in other cognitive tests, including the MMSE [68]. These results are consistent with our observation that dietary and erythrocyte *n*-3 VLCFA were associated with executive function, but not with MMSE or memory pattern scores. The MMSE is considered to be primarily a memory and language-based instrument [69], and it may not adequately capture variation in executive function [70,71]. In a study of healthy elderly adults, baseline plasma EPA plus DHA was protective against cognitive decline as measured with the Trail Making Test Part B score, but not with the MMSE score, over a 4-year follow-up [72]. Tests of verbal fluency appear to contain a large executive functioning component [73] and in a study of middle aged adults, both cholesteryl ester and phospholipid *n*-3 compositions were associated with better scores on the Word Fluency Test after 6-year follow-up [74].

Among those considered to be cognitively normal, we found that greater ARA erythrocyte composition was modestly associated with an elevated risk of 2-year cognitive impairment. Few studies have investigated individual circulating or dietary *n*-6 PUFA in relation to cognitive outcomes. Consistent with our findings, higher ARA composition of cholesteryl esters (but not phospholipids) was associated with greater risk of 6-year decline in a composite cognitive function score among adults (50 to 65 years) [74]. We also observed that greater erythrocyte EDA composition was modestly associated with lower global cognitive function and executive function at 2-year follow-up, but LA concentration was not significantly associated with any outcome. These findings highlight the need to consider individual species in the examination of *n*-6 PUFA in relation to health outcomes, as they may have unique effects.

EDA and ARA are products of *n*-6 LA metabolism [51]. ARA is thought to be proinflammatory because it can be metabolized into eicosanoids known to stimulate inflammation [51]. The biological effects of EDA (which represents only 0.31% of erythrocyte FA) are not well characterized, but cell culture evidence suggests that it may affect inflammation through modulation of pro-inflammatory mediators PGE2, COX-2, and NF-kb [75]. Our results suggest that upregulation of ARA metabolism may adversely affect cognitive function. This could result from altered *n*-6 PUFA metabolism or suboptimal *n*-3 PUFA consumption. N-3 and *n*-6 PUFA metabolism are dependent upon the same set of enzymes, resulting in an inhibitory effect on one another [76]. This association appears less likely due to excess *n*-6 consumption, as neither dietary LA or ARA were associated with any cognitive outcome measured. Indeed, ARA supplementation appears to not adversely affect circulating inflammatory makers [51] and LA consumption to not elevate ARA concentration [77]. Considering evidence that circulating *n*-6 PUFA have been shown to be beneficially associated with dementia risk factors, including coronary heart disease [28,29] and diabetes [27], and the lack of cognitive function studies considering individual *n*-6 PUFA, additional prospectively designed studies investigating individual dietary and erythrocyte *n*-6 PUFA are needed to confirm our findings.

A major strength of this study is the use of a prospective design that reduces the opportunity for reverse causation. Examining individual PUFA from the diet and blood samples allowed for a better understanding of the associations of diet and PUFA metabolism on cognitive function. The rich data available in the BPRHS allowed for the statistical control of a number of confounders, but residual confounding remains a concern. The follow-up period of 2 years is a limitation. A longer study period would have allowed for greater decline in cognitive function, improving our ability to detect a FA connection. A clinical diagnosis of dementia or probable Alzheimer disease was not obtained in the BPRHS. In addition, *n*-3 PUFA concentrations were low in this population, with very few individuals (1.4%) having an omega-3 index value in the desirable range (>8–12%) [54]. Hence the null associations observed between *n*-3 PUFA (erythrocyte composition and dietary intake) and the 2-year MMSE score and cognitive impairment may, in part, be due to the failure to achieve threshold concentrations. Lastly, although prospective in design, the observational nature of this study prevents us from drawing causal inferences. 

## 5. Conclusions

In this prospective study of older Puerto Rican adults living on the U.S. mainland, higher dietary intake, of *n*-3 VLCFA, as well as erythrocyte concentrations, were associated with better 2-year executive function, whereas erythrocyte *n*-6 EDA composition was adversely related to executive function and MMSE scores. Erythrocyte ARA was associated with greater incidence of cognitive impairment. Our results suggest that dietary consumption of *n*-3 VLCFA may have a positive impact on executive function. Further, these findings provide some evidence that *n*-6 metabolism favoring greater ARA bioavailability, but not necessarily dietary intake, could increase the risk of cognitive impairment.

## Figures and Tables

**Table 1:** Baseline sample characteristics by quartile of erythrocyte PUFA composition (*n* = 1032).

Characteristics	Quartile of Erythrocyte PUFA Composition				*P*-Trend
	1	2	3	4	
***n*-3 PUFA, % total FA**	5.0 ± 0.8	6.1 ± 0.2	6.8 ± 0.2	8.4 ± 1.2	
Age, year	55.5 ± 0.5	55.4 ± 0.5	57.6 ± 0.5	59.7 ± 0.5	<0.0001
Female, %	73.8	72.4	74.4	70.4	0.52
High school educational attainment or greater	30.0	35.1	32.0	44.4	0.003
BMI, kg/m^2^	32.0 ± 0.4	32.0 ± 0.4	32.2 ± 0.4	31.6 ± 0.4	0.66
Physical activity score	31.2 ± 0.3	31.8 ± 0.3	31.3 ± 0.3	32.1 ± 0.3	0.09
CES-D score	21.8 ± 0.8	19.2 ± 0.8	20.2 ± 0.8	18.0 ± 0.8	0.004
Healthy Eating Index 2005 score	69.4 ± 0.6	71.0 ± 0.6	71.7 ± 0.6	74.7 ± 0.6	<0.0001
Healthy Eating Index 2005 score (excludes healthy oil component)	60.5 ± 0.5	61.8 ± 0.5	62.7 ± 0.5	65.7 ± 0.5	<0.0001
Currently smoking, %	30.9	28.6	19.5	15.2	<0.0001
Diabetes, %	36.4	39	40.2	40.5	0.32
Cardiovascular disease, %	17	22.4	18.2	26.6	0.03
*n*-3 supplement use, %	0.76	0.77	1.92	7.4	<0.0001
***n*-6 PUFA, % total FA**	32.4 ± 0.3	35.3 ± 0.4	36.6 ± 0.3	38.1 ± 0.9	
Age, year	58.6 ± 0.5	58.1 ± 0.5	56.2 ± 0.5	55.3 ± 0.5	<0.0001
Female, %	68.7	69.1	73.9	79.4	0.003
High school educational attainment or greater	39.9	37.7	30.8	33	0.04
BMI, kg/m^2^	31.5 ± 0.4	32.0 ± 0.4	32.6 ± 0.4	31.7 ± 0.4	0.48
Physical activity score	31.6 ± 0.3	31.4 ± 0.3	31.2 ± 0.3	32.2 ± 0.3	0.27
CES-D score	19.7 ± 0.8	20.0 ± 0.8	19.7 ± 0.8	19.9 ± 0.8	0.88
Healthy Eating Index 2005 score	73.0 ± 0.6	71.3 ± 0.6	71.8 ± 0.6	70.7 ± 0.6	0.01
Healthy Eating Index 2005 score (excludes healthy oil component)	64.2 ± 0.5	62.3 ± 0.5	62.6 ± 0.5	61.5 ± 0.5	0.001
Currently smoking, %	21.5	25.8	21.8	25.1	0.54
Diabetes, %	45.5	37.3	41.6	31.7	0.006
Cardiovascular disease, %	23.7	22.5	21.9	16	0.39
*n*-3 supplement use, %	7.32	0.78	1.96	0.77	<0.0001

CES-D, Center for Epidemiologic Studies Depression Scale; FA, fatty acid; PUFA, polyunsaturated fatty acid. Data represent means ± SD or proportion. Age comparisons are adjusted for sex and those for sex by age. All other comparisons are age- and sex-adjusted.

**Table 2:** Associations of baseline *n*-3 PUFA erythrocyte composition and dietary intake (quartiles) with 2-year executive function score, adjusted for baseline score ^a,b^.

PUFA	MMSE Score, *n* = 1032					Executive Function Score, *n* = 865				
	PUFA Quartile				*P*-Trend	PUFA Quartile				*P*-Trend
	1	2	3	4		1	2	3	4	
**Erythrocyte**										
ALA	23.3 (22.9, 23.6)	23.3 (22.9, 23.6)	23.5 (23.1, 23.8)	23.6 (23.3, 24)	0.07	0.056 (−0.039, 0.15)	0.062 (−0.027, 0.15)	0.042 (−0.05, 0.13)	0.12 (0.031, 0.22)	0.34
EPA	23.3 (22.9, 23.6)	23.5 (23.1, 23.8)	23.5 (23.1, 23.8)	23.4 (23.1, 23.8)	0.52	0.057 (−0.034, 0.15)	0.056 (−0.036, 0.15)	0.048 (−0.043, 0.14)	0.13 (0.034, 0.22)	0.29
DPA	23.4 (23, 23.8)	23.4 (23, 23.7)	23.4 (23.1, 23.8)	23.5 (23.1, 23.8)	0.73	0.078 (−0.015, 0.17)	0.08 (−0.01, 0.17)	0.075 (−0.015, 0.17)	0.053 (−0.041, 0.15)	0.68
DHA	23.2 (22.9, 23.6)	23.5 (23.2, 23.9)	23.5 (23.1, 23.8)	23.3 (23, 23.7)	0.64	0.04 (−0.052, 0.13)	0.012 (−0.078, 0.1)	0.12 (0.032, 0.21)	0.12 (0.027, 0.22)	0.06
*n*-3 VLCFA	23.2 (22.9, 23.6)	23.6 (23.2, 23.9)	23.3 (23, 23.7)	23.5 (23.1, 23.8)	0.55	0.016 (−0.077, 0.11)	0.053 (−0.036, 0.14)	0.046 (−0.045, 0.14)	0.17 (0.076, 0.27) ^†^	0.02
**Diet**										
ALA	23.3 (23, 23.7)	23.3 (23, 23.7)	23.6 (23.2, 23.9)	23.5 (23.1, 23.8)	0.31	0.064 (−0.029, 0.16)	0.058 (−0.033, 0.15)	0.078 (−0.014, 0.17)	0.1 (0.0094, 0.19)	0.50
EPA	23.2 (22.9, 23.6)	23.4 (23.1, 23.8)	23.3 (23, 23.7)	23.7 (23.3, 24)	0.09	0.032 (−0.063, 0.13)	0.027 (−0.064, 0.12)	0.026 (−0.062, 0.11)	0.22 (0.13, 0.32) *	0.004
DPA	23.2 (22.9, 23.6)	23.4 (23.1, 23.8)	23.4 (23, 23.7)	23.6 (23.3, 24)	0.19	0.05 (−0.048, 0.15)	−0.0094 (−0.1, 0.083)	0.059 (−0.032, 0.15)	0.21 (0.1, 0.31)	0.02
DHA	23.2 (22.9, 23.6)	23.4 (23.1, 23.8)	23.4 (23.1, 23.8)	23.6 (23.2, 24)	0.19	0.033 (−0.064, 0.13)	−0.021 (−0.11, 0.069)	0.057 (−0.032, 0.15)	0.23 (0.14, 0.33) *	0.0009
*n*-3 VLCFA	23.2 (22.9, 23.6)	23.3 (23, 23.7)	23.4 (23.1, 23.8)	23.7 (23.3, 24)	0.08	0.032 (−0.065, 0.13)	−0.0076 (−0.099, 0.083)	0.037 (−0.053, 0.13)	0.24 (0.14, 0.33) *	0.001

^a^ ARA, arachidonic; ALA, α-linolenic; DGLA, dihomo-γ-linolenic acid; DHA, docosahexaenoic; DPA, docosapentaenoic; DTA, docosatetraenoic; EDA, eicosadienoic; EPA, eicosapentaenoic; GED, General Education Development certificate; GLA, γ-linolenic acid; LA, linoleic acid; MMSE, Mini-Mental State Exam; PUFA, polyunsaturated fatty acid; VLCFA, very-long-chain fatty acid; ^b^ Models are adjusted for time between visits and sex and baseline values for MMSE score or executive function score, age, education (≥high school/GED or not), BMI, physical activity score, depressive symptomatology (CES-D score ≥16 or not), HEI 2005 score (excluding healthy oil component score), smoking status, diabetes, and heart disease. Erythrocyte *n*-3 models were also adjusted for erythrocyte ARA and LA composition, whereas dietary *n*-3 models were adjusted for dietary consumption of ARA and LA. * *P* < 0.05; ^†^ *P* = 0.06 compared to quartile 1.

**Table 3:** Associations of *n-*6 PUFA erythrocyte composition and dietary intake (quartiles) with 2-year MMSE and executive function scores, adjusted for baseline scores ^a,b^.

PUFA	MMSE Score, *n* = 1032					Executive Function Score, *n* = 865				
	PUFA Quartile				*P*-Trend	PUFA Quartile				*P*-Trend
	1	2	3	4		1	2	3	4	
**Erythrocyte**										
LA	23.4 (23, 23.7)	23.7 (23.3, 24.1)	23.4 (23.1, 23.8)	23.2 (22.8, 23.6)	0.27	0.1 (0.013, 0.19)	0.089 (−0.0019, 0.18)	0.054 (−0.037, 0.14)	0.044 (−0.053, 0.14)	0.28
GLA	23.4 (23.1, 23.8)	23.2 (22.9, 23.6)	23.3 (22.9, 23.6)	23.8 (23.4, 24.2)	0.09	0.094 (0.0058, 0.18)	0.054 (−0.037, 0.15)	0.064 (−0.029, 0.16)	0.083 (−0.011, 0.18)	0.89
EDA	23.8 (23.5, 24.1)	23.2 (22.9, 23.6) *	23.4 (23, 23.7)	23.2 (22.8, 23.5) *	0.02	0.14 (0.055, 0.23)	0.09 (−0.00077, 0.18)	0.015 (−0.078, 0.11)	0.031 (−0.062, 0.12)	0.02
DGLA	23.4 (23.1, 23.8)	23.4 (23.1, 23.8)	23.4 (23.1, 23.8)	23.4 (23.1, 23.8)	0.97	0.083 (−0.0079, 0.17)	0.064 (−0.025, 0.15)	0.092 (0.0011, 0.18)	0.06 (−0.033, 0.15)	0.81
ARA	23.6 (23.2, 23.9)	23.3 (23, 23.6)	23.3 (23, 23.7)	23.5 (23.1, 23.8)	0.62	0.063 (−0.029, 0.16)	0.085 (−0.0042, 0.17)	0.082 (−0.0092, 0.17)	0.068 (−0.027, 0.16)	0.96
DTA	23.5 (23.1, 23.8)	23.6 (23.2, 23.9)	23.4 (23.1, 23.8)	23.3 (22.9, 23.6)	0.32	0.13 (0.035, 0.23)	0.066 (−0.025, 0.16)	0.065 (−0.024, 0.15)	0.045 (−0.048, 0.14)	0.19
DPA	23.5 (23.2, 23.9)	23.4 (23, 23.7)	23.4 (23.1, 23.8)	23.4 (23, 23.7)	0.57	0.08 (−0.014, 0.17)	0.061 (−0.031, 0.15)	0.036 (−0.053, 0.13)	0.12 (0.026, 0.21)	0.67
**Diet**										
LA	23.5 (23.1, 23.8)	23.5 (23.2, 23.9)	23.4 (23.1, 23.8)	23.3 (22.9, 23.6)	0.36	0.063 (−0.029, 0.16)	0.079 (−0.011, 0.17)	0.1 (0.014, 0.19)	0.046 (−0.048, 0.14)	0.89
ARA	23.4 (23.1, 23.8)	23.5 (23.2, 23.9)	23.3 (23, 23.7)	23.4 (23, 23.8)	0.74	0.035 (−0.059, 0.13)	0.058 (−0.033, 0.15)	0.13 (0.037, 0.22)	0.072 (−0.023, 0.17)	0.38

^a^ ARA, arachidonic; ALA, α-linolenic; DGLA, dihomo-γ-linolenic acid; DHA, docosahexaenoic; DPA, docosapentaenoic; DTA, docosatetraenoic; EDA, eicosadienoic; EPA, eicosapentaenoic; GED, General Education Development certificate; GLA, γ-linolenic acid; LA, linoleic acid; MMSE, Mini-Mental State Exam; PUFA, polyunsaturated fatty acid; VLCFA, very-long-chain fatty acid; ^b^ Models are adjusted for time between visits and sex and baseline values for MMSE score or executive function score, age, education (≥high school/GED or not), BMI, physical activity score, depressive symptomatology (CES-D score ≥16 or not), HEI 2005 score (excluding healthy oil component score), smoking status, diabetes, and heart disease. Erythrocyte *n*-6 models were also adjusted for erythrocyte ALA and *n*-3 VLCFA composition, whereas dietary *n*-6 models were adjusted for dietary consumption of ALA and *n*-3 VLCFA. * *P* < 0.05 compared to quartile 1.

**Table 4:** Association between baseline erythrocyte *n*-3 PUFA composition (z-score) and risk of 2-year cognitive impairment among those considered to not have cognitive impairment at baseline (*n* = 688) ^a,b,c^.

PUFA	OR (CI)	*P*-Value
**Erythrocyte**		
ALA	0.96 (0.80, 1.15)	0.64
EPA	0.96 (0.70, 1.33)	0.82
DPA	0.95 (0.81, 1.11)	0.50
DHA	0.90 (0.76, 1.07)	0.23
*n*-3 VLCFA	0.89 (0.74, 1.07)	0.23
**Diet**		
ALA	0.93 (0.80, 1.09)	0.37
EPA	0.92 (0.77, 1.10)	0.37
DPA	0.94 (0.77, 1.16)	0.57
DHA	0.90 (0.73, 1.10)	0.30
*n*-3 VLCFA	0.91 (0.74, 1.10)	0.33

^a^ ARA, arachidonic; ALA, α-linolenic; DGLA, dihomo-γ-linolenic acid; DHA, docosahexaenoic; DPA, docosapentaenoic; DTA, docosatetraenoic; EDA, eicosadienoic; EPA, eicosapentaenoic; GLA, γ-linolenic acid; LA, linoleic acid; MMSE, Mini-Mental Sate Exam; OR, Odds Ratio; PUFA, polyunsaturated fatty acid; VLCFA, very-long-chain fatty acid; ^b^ 688 individuals of 1032 (67%) were considered to not have cognitive impairment at baseline based on MMSE and education level specific cut-offs. Erythrocyte PUFA are expressed as z-scores. OR can be interpreted as the risk of 2-y cognitive impairment for a 1-SD change in erythrocyte PUFA; ^c^ Models are adjusted for time between visits and sex and baseline values for BMI, physical activity score, depressive symptomatology (CES-D score ≥16 or not), HEI 2005 score (excluding healthy oil component score), smoking status, diabetes, and heart disease. Erythrocyte *n*-3 models were also adjusted for erythrocyte ARA and LA composition, whereas dietary *n*-3 models were adjusted for dietary consumption of ARA and LA.

**Table 5:** Association between baseline erythrocyte *n*-6 PUFA composition (z-score) and risk of 2-year cognitive impairment among those considered to not have cognitive impairment at baseline (*n* = 688) ^a,b,c^.

PUFA	OR (CI)	*P*-Value
**Erythrocyte**		
LA	1.04 (0.88, 1.22)	0.65
GLA	0.92 (0.52, 1.62)	0.77
EDA	1.03 (0.90, 1.18)	0.69
DGLA	0.98 (0.85, 1.13)	0.81
ARA	1.26 (1.05, 1.50)	0.01
DTA	1.13 (0.96, 1.33)	0.13
DPA	1.10 (0.95, 1.28)	0.20
**Diet**		
LA	0.96 (0.83, 1.12)	0.60
ARA	0.89 (0.75, 1.06)	0.19

^a^ ARA, Arachidonic; ALA, α-Linolenic; DGLA, Dihomo-year-linolenic; DHA, Docosahexaenoic; DPA, Docosapentaenoic; DTA, Docosatetraenoic; EDA, Eicosadienoic; EPA, Eicosapentaenoic; GLA, γ-Linolenic; LA, Linoleic; MMSE, Mini-Mental Sate Exam; OR, Odds Ratio; PUFA, polyunsaturated fatty acid; VLCFA, very-long-chain fatty acid; ^b^ 688 individuals of 1032 (67%) were considered to not have cognitive impairment at baseline based on MMSE and education level specific cut-points. Erythrocyte PUFA are expressed as z-scores. OR can be interpreted as the risk of 2-year cognitive impairment for a 1-SD change in erythrocyte PUFA; ^c^ Models are adjusted for time between visits and sex and baseline values for BMI, physical activity score, depressive symptomatology (CES-D score ≥16 or not), HEI 2005 score (excluding healthy oil component score), smoking status, diabetes, and heart disease. Erythrocyte *n*-6 models were also adjusted for erythrocyte ALA and *n*-3 VLCFA composition, whereas dietary *n*-6 models were adjusted for dietary consumption of ALA and *n*-3 VLCFA.

## Supplementary Material

The following are available online at www.mdpi.com/xxx/s1; Table S1: Coefficients of variation of erythrocyte fatty acid composition; Table S2: Unadjusted mean (5th and 95th percentile) values of erythrocyte and dietary PUFA by quartile, *n* = 1032; Table S3: Factor loadings of two cognitive testing patterns among Puerto Rican Adults; Table S4: Baseline sample characteristics by quartile of dietary fatty acid composition, *n* = 1032; Table S5: Correlations between erythrocyte and dietary PUFA species, *n* = 1032; Table S6: Baseline sample characteristics by baseline cognitive impairment status, *n* = 1032.
