# Supplementary material for: Prospective Associations of Erythrocyte Composition and Dietary Intake of n-3 and n-6 PUFA with Measures of Cognitive Function
Source: Nutrients. 2018 Sep 6;10(9):1253. doi: 10.3390/nu10091253 (PMC6164488; doi:10.3390/nu10091253)
Supplement: Supplementary file 1 [file nutrients-10-01253-s001.pdf]

Table S1. Coefficients of variation of erythrocyte fatty acid composition

Fatty acid		Coefficient of variation
Linoleic	C18:2n6	12.3%
γ-Linolenic	C18:3n6	0.15%
α-Linolenic	C18:3n3	0.13%
Eicosadienoic	C20:2n6	0.31%
Dihomo-y-linolenic	C20:3n6	1.83%
Arachidonic	C20:4n6	16.6%
Eicosapentaenoic	C20:5n3	0.43%
Docosatetraenoic	C22:4n6	3.70%
Docosapentaenoic	C22:5n6	0.79%
Docosapentaenoic	C22:5n3	2.00%
Docosahexaenoic	C22:6n3	3.95%

Values are only presented for *n*-3 and *n*-6 polyunsaturated fatty acids

Table S2. Unadjusted mean (5th and 95th percentile) values of erythrocyte and dietary PUFA by quartile, n=1032

	PUFA Quartile			
	**1**	**2**	**3**	**4**
n-3 PUFA				
Erythrocyte, % total FA				
ALA	0.072	0.11	0.14	0.21
	(0.043 ,0.091)	(0.094 ,0.12)	(0.12 ,0.16)	(0.16 ,0.34)
EPA	0.24	0.34	0.44	0.78
	(0.14 ,0.3)	(0.3 ,0.38)	(0.39 ,0.49)	(0.51 ,1.4)
DPA	1.6	1.9	2.1	2.5
	(1.1 ,1.8)	(1.8 ,2)	(2 ,2.2)	(2.2 ,2.9)
DHA	2.7	3.6	4.2	5.4
	(1.8 ,3.2)	(3.3 ,3.9)	(3.9 ,4.6)	(4.7 ,6.8)
n-3 VLCFA	4.9	6	6.7	8.3
	(3.4 ,5.5)	(5.6 ,6.3)	(6.4 ,7.1)	(7.2 ,11)
Diet, % energy				
ALA	0.41	0.51	0.6	0.79
	(0.31 ,0.46)	(0.47 ,0.54)	(0.55 ,0.65)	(0.66 ,1.1)
EPA	0.0079	0.017	0.029	0.072
	(0.0031 ,0.012)	(0.013 ,0.021)	(0.022 ,0.036)	(0.038 ,0.18)
DPA	0.0041	0.0074	0.011	0.022
	(0.0019 ,0.0058)	(0.0061 ,0.0089)	(0.0093 ,0.014)	(0.014 ,0.044)
DHA	0.019	0.036	0.058	0.13
	(0.0085 ,0.026)	(0.028 ,0.045)	(0.047 ,0.072)	(0.075 ,0.27)
n-3 VLCFA	0.032	0.061	0.098	0.22
	(0.015 ,0.046)	(0.048 ,0.075)	(0.079 ,0.12)	(0.13 ,0.48)
n-6 PUFA				
Erythrocyte, % total FA				
LA	10	11.6	12.8	14.8
	(8.57 ,11)	(11.2 ,12.2)	(12.2 ,13.5)	(13.6 ,17.3)
GLA	0.11	0.14	0.16	0.27
	(0.078 ,0.12)	(0.13 ,0.15)	(0.15 ,0.17)	(0.18 ,0.28)
EDA	0.25	0.29	0.33	0.39
	(0.21 ,0.28)	(0.28 ,0.31)	(0.31 ,0.34)	(0.35 ,0.46)
DGLA	1.4	1.7	1.9	2.4
	(1.1 ,1.5)	(1.6 ,1.8)	(1.8 ,2)	(2.1 ,2.9)
ARA	14.2	16.1	17.2	18.5
	(11.3 ,15.5)	(15.6 ,16.6)	(16.6 ,17.6)	(17.8 ,19.8)
DTA	2.8	3.5	3.9	4.5
	(1.8 ,3.3)	(3.3 ,3.7)	(3.7 ,4.1)	(4.2 ,5.2)
DPA	0.56	0.72	0.83	1
	(0.37 ,0.66)	(0.67 ,0.77)	(0.78 ,0.89)	(0.9 ,1.3)
Diet, % energy				
LA	5.4	7.3	8.6	10
	(3.8 ,6.5)	(6.7 ,7.9)	(8 ,9.1)	(9.3 ,12)
ARA	0.033	0.05	0.066	0.1
	(0.016 ,0.043)	(0.044 ,0.057)	(0.059 ,0.076)	(0.077 ,0.14)

ARA, Arachidonic; ALA, α-Linolenic; DGLA, Dihomo-y-linolenic; DHA, Docosahexaenoic; DPA, Docosapentaenoic; DTA, Docosatetraenoic; EDA, Eicosadienoic; EPA, Eicosapentaenoic; GLA, γ-Linolenic; LA, Linoleic; PUFA, polyunsaturated fatty acid; VLCFA, very-long-chain fatty acid (EPA+DPA+DHA).

Table S3. Factor loadings of two cognitive testing patterns among Puerto Rican Adults

Cognitive Testing Variable	Description of test	Mean SD	Min-Max	Factor1 Executive Function	Factor2 Memory
Word-list learning test: Immediate Recall	A list of words is read and participants gets 5 attempts to repeat the list of words. Total number of correct words at each attempt are summed.	38.4 ± 10.3	4 - 71		0.75
Word-list learning test: Retention and recognition	A list of words is read and participant identifies those words that were read from a previous word list	29.7 ± 5.7	0 - 36.4		0.83
Verbal fluency test	Name as many words possible starting with letter C. Repeat with letters F and L. Score calculated by summing number of words named in each trial.	24.5 ± 9.9	0-65	0.61	
Figure copying	Draw 8 figures. Each figure is scored for accuracy and a sum total score calculated. Score is weighted for difficulty of figure.	11.1 ± 7.4	0 - 27	0.66	
Digit span forward	Read 2 sequences of numbers and participants repeats list from first to last.	7.4 ± 1.9	0 - 15	0.59	
Digit span backward	Read 2 sequences of numbers and participants repeats list from last to first.	3.5 ± 1.5	0 - 9	0.66	
Clock drawing	Draw a clock with a specified time.	2.1 ± 0.9	0 - 3	0.57	
Stroop test 1	Read the list of words (colors) as fast as possible in 45 seconds. Number of correctly read words is added.	70.5 ± 20.3	0 - 135	0.61	0.38
Stroop test 2	Read the list of words (colors) as fast as possible in 45 seconds. Number of correctly read words is added.	46.8 ± 14.8	0-102	0.66	0.46
Stroop test 3	Name the ink color that each word (color) is written in 45 seconds. Number of correctly read words is added.	23.7 ± 10.3	0-68	0.63	0.38

Factor loadings of >0.35 are shown.

Table S4. Baseline sample characteristics by quartile of dietary fatty acid composition, n=1032

Characteristics	Quartile of Dietary PUFA Consumption				*P*-Trend
	**1**	**2**	**3**	**4**	
***n*-3 PUFA, % energy**	0.47 ± 0.06	0.59 ± 0.03	0.71 ± 0.04	0.94 ± 0.14	
Age, y	57.6 ± 0.468	56.5 ± 0.468	56.9 ± 0.468	57.2 ± 0.468	0.74
Female, %	70.8	69.5	74.8	75.9	0.10
High school educational attainment or greater	34.6	37	34.7	35.3	0.99
BMI, kg/m^2	31.4 ± 0.408	31.8 ± 0.408	32.2 ± 0.408	32.4 ± 0.408	0.09
Physical activity score	31.6 ± 0.285	31.8 ± 0.285	31.4 ± 0.285	31.6 ± 0.285	0.79
CES-D score	21.2 ± 0.796	18.8 ± 0.796	18.6 ± 0.796	20.7 ± 0.796	0.63
Healthy Eating Index 2005 score	68.1 ± 0.548	72.2 ± 0.548	72.5 ± 0.548	74.1 ± 0.548	<0.0001
Healthy Eating Index 2005 score (excludes healthy oil component) hei2005_	59.9 ± 0.534	63.2 ± 0.534	63.1 ± 0.534	64.4 ± 0.534	<0.0001
Currently smoking, %	28.2	23.2	19.8	23	0.10
Diabetes, %	35.8	34.4	41.3	44.7	0.01
Cardiovascular disease, %	17.8	20.9	18.7	26.7	0.03
n-3 supplement use, %	1.52	0.836	3.1	5.4	0.002
***n*-6 PUFA, % energy**	5.5 ± 0.84	7.4 ± 0.42	8.6 ± 0.35	10.5 ± 1.04	
Age, y	58.7 ± 0.464	57 ± 0.464	56.4 ± 0.464	56.1 ± 0.464	<0.0001
Female, %	73	72.5	71.4	74.2	0.84
High school educational attainment or greater	41.1	37	35.3	28	0.002
BMI, kg/m^2	32.1 ± 0.41	31.5 ± 0.41	32.1 ± 0.41	32.2 ± 0.41	0.55
Physical activity score	31.8 ± 0.286	31.8 ± 0.286	31.6 ± 0.286	31.2 ± 0.286	0.12
CES-D score	20 ± 0.802	19.8 ± 0.802	18.8 ± 0.802	20.6 ± 0.802	0.84
Healthy Eating Index 2005 score	69.8 ± 0.562	71.5 ± 0.562	73.2 ± 0.562	72.4 ± 0.562	0.001
Healthy Eating Index 2005 score (excludes healthy oil component) HE	62.7 ± 0.546	62.2 ± 0.546	63.4 ± 0.546	62.4 ± 0.546	0.96

Currently smoking, %	22	22.4	19.8	30	0.07
Diabetes, %	35.6	38.5	41.5	40.6	0.19
Cardiovascular disease, %	22.1	20.2	22.9	18.9	0.54
n-3 supplement use, %	4.96	2.72	2.37	0.809	0.01

CES-D, Center for Epidemiologic Studies Depression Scale; PUFA, Polyunsaturated Fatty Acid. Data represent means ± SD or proportion. Age comparisons are adjusted for sex and those for sex by age. All other comparisons are age- and sex-adjusted.

Table S5. Correlations between erythrocyte and dietary PUFA species, n=1032

		Erythrocyte, % total FA											Diet, % energy					
		***n*-3 PUFA**				***n*-6 PUFA**							***n*-3 PUFA**				***n*-6 PUFA**	
		**ALA**	**EPA**	**DPA**	**DHA**	**LA**	**GLA**	**EDA**	**DGLA**	**ARA**	**DTA**	**DPA**	**ALA**	**EPA**	**DPA**	**DHA**	**LA**	**ARA**
Erythrocyte, % total FA																		
n-3 PUFA	ALA	--	0.35*	0.21*	0.03	0.31*	0.23*	-0.07*	0.14*	-0.34*	-0.34*	-0.28*	0.12*	-0.08*	0.07*	0.10*	-0.11*	0.02
	EPA	--	--	0.60*	0.44*	-0.19*	0.26*	-0.37*	0.01	-0.04	-0.41*	-0.45*	0.10*	0.28*	0.25*	0.28*	-0.14*	0.07*
	DPA	--	--	--	0.19*	-0.22*	0.10*	-0.23*	0.02	0.14*	0.02	-0.23*	0.04	0.03	0.05	0.03	-0.12*	-0.08*
	DHA	--	--	--	--	-0.20*	-0.18*	-0.15*	-0.09*	0.04	-0.23*	-0.11*	0.04	0.38*	0.34*	0.38*	-0.05	0.15*
n-6 PUFA	LA	--	--	--	--	--	-0.02	0.41*	0.22*	-0.43*	-0.21*	-0.19*	0.06*	0.01	0.01	0.01	0.03	-0.01
	GLA	--	--	--	--	--	--	-0.13*	0.08*	0.02	-0.04	-0.10*	0.05	-0.05	-0.07*	-0.04	-0.01	-0.06*
	EDA	--	--	--	--	--	--	--	0.21*	-0.17*	0.08*	0.09*	0.08*	-0.08*	-0.07*	-0.06	0.12*	0.003
	DGLA	--	--	--	--	--	--	--	--	-0.34*	-0.14*	0.11*	-0.05	-0.08*	-0.03	-0.05	-0.06	-0.01
	ARA	--	--	--	--	--	--	--	--	--	0.46*	0.37*	-0.02	-0.09*	-0.04	-0.10*	0.09*	0.03
	DTA	--	--	--	--	--	--	--	--	--	--	0.52*	-0.04	-0.28*	-0.22*	-0.28*	0.14*	-0.07*
	DPA	--	--	--	--	--	--	--	--	--	--	--	-0.13*	-0.22*	-0.19*	-0.21*	0.05	0.04
Diet, % energy																		
n-3 PUFA	ALA	--	--	--	--	--	--	--	--	--	--	--	--	0.19*	0.19*	0.24*	0.43*	0.23*
	EPA	--	--	--	--	--	--	--	--	--	--	--	--	--	0.84*	0.93*	0.08*	0.44*
	DPA	--	--	--	--	--	--	--	--	--	--	--	--	--	--	0.87*	0.18*	0.63*
	DHA	--	--	--	--	--	--	--	--	--	--	--	--	--	--	--	0.12*	0.53*
n-6 PUFA	LA	--	--	--	--	--	--	--	--	--	--	--	--	--	--	--	--	0.31*
	ARA	--	--	--	--	--	--	--	--	--	--	--	--	--	--	--	--	--

ARA, Arachidonic; ALA, α-Linolenic; DGLA, Dihomo-y-linolenic; DHA, Docosahexaenoic; DPA, Docosapentaenoic; DTA, Docosatetraenoic; EDA, Eicosadienoic; EPA, Eicosapentaenoic; GLA, γ-Linolenic; LA, Linoleic; PUFA, polyunsaturated fatty acid. Data are age, sex, and energy adjusted Spearman's correlations coefficients. *$P < 0.05$.

Table S6. Baseline sample characteristics by baseline cognitive impairment status, n=1032

	Categorized as having cognitive impairment		*P*-Value
	No	Yes	
	n=688	n=344	
Mini-Mental State Exam	25.1 ± 0.07	20.0 ± 0.10	<0.0001
Age, y	56.5 ± 0.29	58.1 ± 0.285	0.001
Female, %	70.7	76.9	0.04
High school educational attainment or greater	36.3	33.5	0.36
BMI, kg/m^2	31.9 ± 0.25	32.1 ± 0.25	0.67
Physical activity score	31.9 ± 0.17	31 ± 0.174	0.002
CES-D score	18.6 ± 0.485	22.3 ± 0.485	<0.0001
Healthy Eating Index 2005 score	72.3 ± 0.345	70.5 ± 0.345	0.003
Healthy Eating Index 2005 score (excludes healthy oil component)	63.2 ± 0.332	61.5 ± 0.332	0.003
Currently smoking, %	23.4	23.8	0.90
Diabetes, %	38.1	40.9	0.38
Cardiovascular disease, %	20.9	21.4	0.85
n-3 supplement use, %	3.41	1.32	0.05

Data represent means ± SD or proportions. Age comparisons are adjusted for sex and those for sex by age. Other comparisons are age- and sex-adjusted and, for Mini-Mental State Exam, additionally adjusted for high school educational attainment or greater. Presence of cognitive impairment was defined as an MMSE score <21 for those with less than a high school education or General Education Equivalency, <23 for those that completed high school, and <24 for those with some college education.
